# Regional Coronavirus Hotspots During the COVID-19 Outbreak in the Netherlands

**DOI:** 10.1007/s10645-021-09383-4

**Published:** 2021-04-21

**Authors:** Wolter H. J. Hassink, Guyonne Kalb, Jordy Meekes

**Affiliations:** 1 Utrecht University School of Economics, Utrecht University, Kriekenpitplein 21-22, 3584 EC Utrecht, The Netherlands; 2 Melbourne Institute: Applied Economic & Social Research, The University of Melbourne, Melbourne, Australia; 3 IZA–Institute of Labor Economics, Bonn, Germany; 4 LCC–The ARC Centre of Excellence for Children and Families over the Life Course, Melbourne, Australia

**Keywords:** COVID-19, Coronavirus hotspots, Lockdown, Employment, Working hours, Wages, I15, I18, J20, J30, J64

## Abstract

We explore the impact of COVID-19 hotspots and regional lockdowns on the Dutch labour market during the outbreak of COVID-19. Using weekly administrative panel microdata for 50 per cent of Dutch employees until the end of March 2020, we study whether individual labour market outcomes, as measured by employment, working hours and hourly wages, were more strongly affected in provinces where COVID-19 confirmed cases, hospitalizations and mortality were relatively high. The evidence suggests that labour market outcomes were negatively affected in all regions and local higher virus case numbers did not reinforce this decline. This suggests that preventive health measures should be at the regional level, isolating hotspots from low-risk areas.

## Introduction

The pandemic that started in 2020 has led to the first world-wide economic downturn in recent times triggered by a deadly virus. As the outbreak of COVID-19 commenced, governments were confronted with the dilemma of how to balance the economic and health costs of a surge in COVID-19 cases versus the costs of preventive health measures to stop the spread of the virus (Layard et al., 2020). Two major complementary mechanisms for how COVID-19 may have affected short-run labour market outcomes are investigated in this paper.

First, there is a direct economic effect arising from the population’s health concerns due to COVID-19, which may differ by location, as people living in so-called COVID-19 hotspot areas may be more aware of the presence and detrimental consequences of the virus. As a result, they would take voluntary preventive measures of social distancing more seriously, which is likely to have a negative impact on social activity and the labour market. Second, the indirect economic effect through (regional) enforced lockdown and social distancing regulations by the government in response to the virus would lead to an immediate loss of the economic activities that are no longer allowed, and a negative impact on labour market outcomes of workers who can be dismissed easily.

This paper assesses the relevance of the above two mechanisms for how the COVID-19 outbreak has affected the labour market in the Netherlands. We use unique Dutch administrative weekly panel microdata covering the period until the end of March 2020, drawing a random sample of 50 per cent of all Dutch employees (about 4.2 million individuals). Taking a regional perspective, we examine whether the economic slowdown as measured by individual labour market outcomes has been stronger in COVID-19 hotspot areas where a substantial proportion of the population was affected by the virus. Such a geographical examination is important as different labour market changes across regions suggest that it may be worthwhile to consider imposing preventative measures, such as a lockdown, at the regional (rather than national) level, isolating hotspots from low-risk areas.^1^ This is illustrated by the Australian case, where the economic recovery after the first lockdown in March–May 2020 was considerable in all states except for Victoria which experienced a second wave of COVID-19 and local ‘hard’ lockdown from July until October 2020 (Kalb, Guillou, and Meekes, 2020).

The labour-market effects during the COVID-19 outbreak have been documented for many countries by a rapidly expanding literature. The general picture that emerges from these studies is that there was a steep decline of the labour market outcomes in the first months of the outbreak. Furthermore, there was an unequal impact across workers– employees with relatively weak employment protection experienced worse labour market outcomes. We report the outcomes for employment.^2^ For the US, the employment-population ratio fell by about 8 percentage points from February to April, where low-wage workers and minority workers were hit hardest (Coibion et al., 2020; Hershbein & Holzer, 2021). In Japan, in May 2020, regular employment declined by 1 per cent; for workers in non-standard jobs the decline was 4–5 per cent (Kikuchi et al., 2020). In Greece, employment had declined by about 12 per cent by June (Betcherman et al., 2020). For the Netherlands, Von Gaudecker, Holler, Janys, Siflinger, and Zimpelmann (2020), who used monthly survey data on 2918 salaried workers until late March 2020 from the Longitudinal Internet studies for the Social Sciences (LISS) panel, found a reduction in total working hours of 11 per cent or 3 h. In Australia, employment declined by about 6.7 per cent from March to May (Borland & Charlton, 2020). In Mexico, the formal job market declined by 5.4 per cent from March to November (Hoehn-Velasco, Silverio-Murillo, and Balmori de la Miyar, 2021).

Evidence on within-country regional differences in COVID-19 impacts on labour market outcomes is more limited.^3^ For South Korea, Aum, Lee, and Shin (2020) investigate regional differences in the COVID-19 impact on the labour market. They use a differences-in-differences estimator to compare the COVID-19 impact for the local area Shincheonji –which experienced a significant COVID-19 outbreak from February 18 onwards without imposing a lockdown– with other areas that did not experience a significant number of COVID-19 cases. Aum et al. (2020) find that a one per thousand increase in infections caused a 2 to 3 per cent drop in local employment in the initial months after the outbreak (up to May 2020). As South Korea has not imposed any lockdowns, they argue that the estimates imply that at most half of the 5 to 6 per cent decrease in employment in the US and UK can be attributed to lockdowns. For the US, Cho, Lee, and Winters (2020) show that the negative impact of the COVID-19 crisis on employment is larger for metropolitan areas, arguing that the local COVID-19 infection rate explains half of the heterogeneity in employment changes across US metropolitan areas.

## Regional Differences in COVID-19 Cases and Preventive Measures

The outbreak of the COVID-19 pandemic has had a common pattern across countries. First, there was a phase of denial by the authorities and the public, downplaying the severity of the outbreak. Second, a substantial outburst of cases occurred in a local region. Governments responded by introducing preventive measures for this local area only. Third, COVID-19 cases spread to other parts of the country. During the outbreak of the virus, most governments started by imposing a regional lockdown, before broadening it to the entire country. Of all policy measures, the compulsory societal lockdown was the most disruptive to the economy, enforcing social distancing, staying at home and working from home rules.

**Fig. 1:**
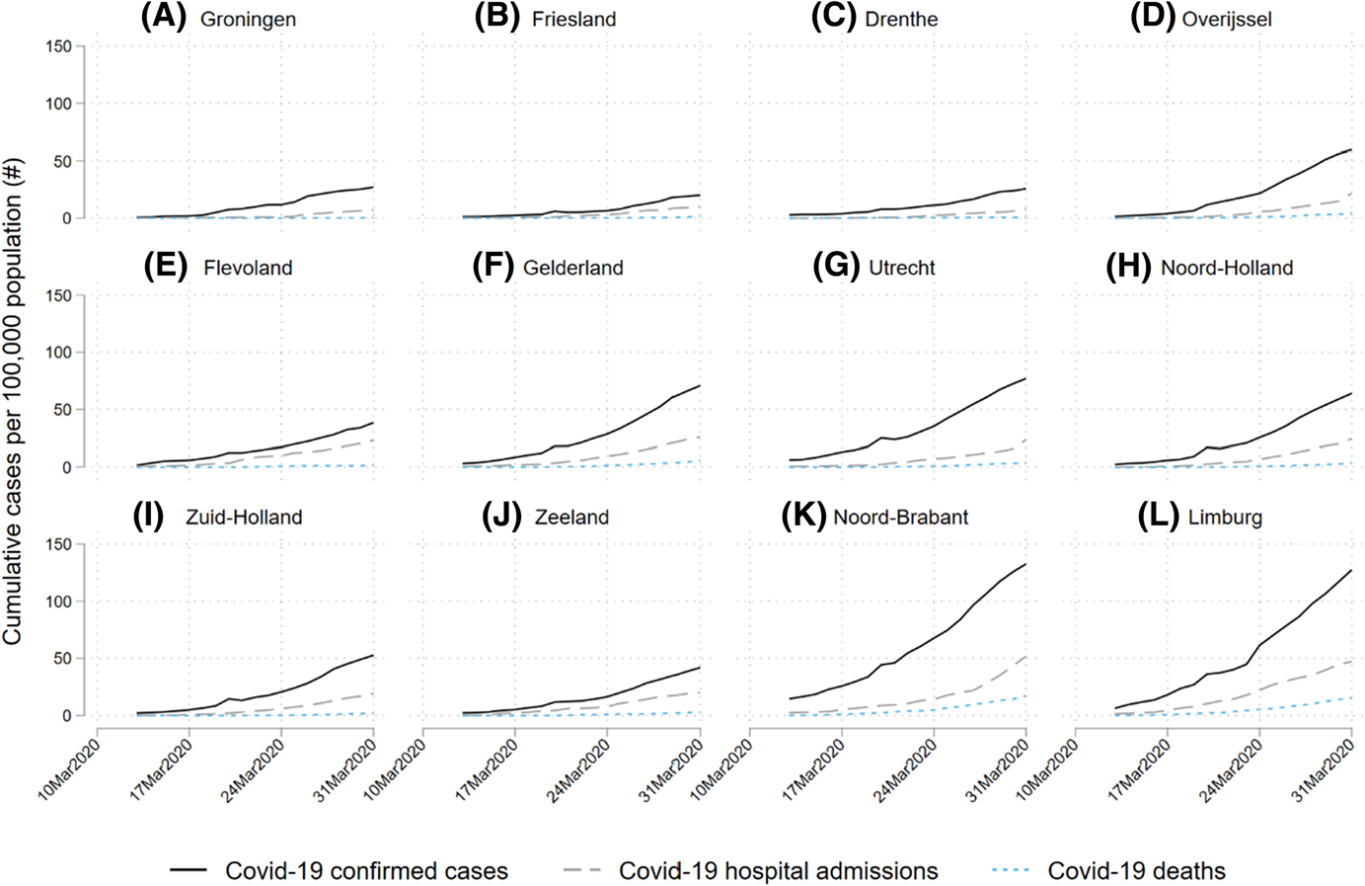
Cumulative number of cases per 100,000 residents by province, March 2020 See Rijksinstituut voor Volksgezondheid en Milieu (RIVM) (2020) for the COVID-19 cases data. See CBS (2020) for the population data

**Fig. 2:**
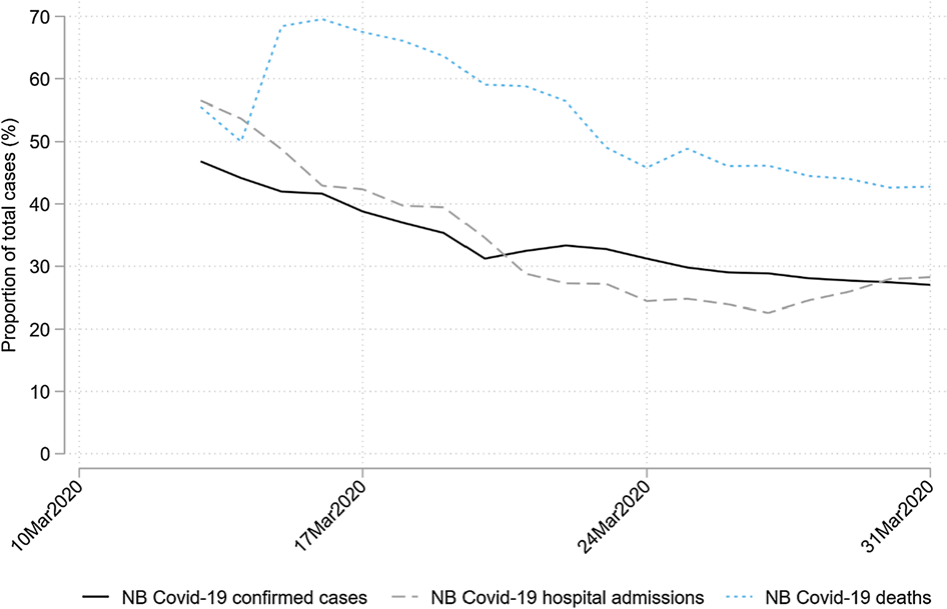
Proportion of cases in Noord-Brabant (NB), relative to the total number of cases in the Netherlands, March 2020 *Notes*: See RIVM (2020) for the COVID-19 cases data. See CBS (2020) for the population data

The virus outbreak in the Netherlands followed this pattern. On February 27 2020, the first person tested positive. In the first weeks of March, the southern province Noord-Brabant had about half of all detected infections in the Netherlands despite this province only accounting for 15 per cent of the Dutch population. At the same time, the northern provinces were almost free of infections. With regard to reported confirmed COVID-19 cases, hospitalizations and deaths, Noord-Brabant was leading in absolute terms per 100,000 residents (Fig. 1) as well as in relative terms as a proportion of total Dutch confirmed COVID-19 cases (Fig. 2). Consequently, the government’s preventive measures were at first directed at Noord-Brabant only. On March 6, people living in this province were advised to stay home, particularly if they had colds, coughs or a fever. On March 9, the Dutch Prime Minister suggested the population of Noord-Brabant should work from home. On March 10, large gatherings were banned in Noord-Brabant. On March 12, restrictions were imposed on the entire country, including social distancing, banning of gatherings over 100 persons, and a work-from-home directive. From March 15 onwards, all restaurants, schools, childcare and sport facilities were closed. On March 23, physical distancing requirements were tightened, imposing the 1.5-m distance measure and cancelling all gatherings including those with fewer than 100 people.

## Statistical Identification

We estimate the causal impact of the COVID-19 outbreak on three individual labour market outcomes: employment, measured by a 0–1 indicator which equals one if the person was employed–at least partly–in a given week; (logarithm of) the number of hours worked; and (logarithm of) the hourly wage.^4^ At the individual level, the information on working hours and gross wages is derived from monthly income statement data. If a calendar week sits across two calendar months, data from the first calendar month is used. Weekly variation in hours worked and hourly wage is driven by changes in employment only (from job to job or to unemployment).

Identification of both mechanisms is based on a specific regional pattern during the virus outbreak in the Netherlands. In the first weeks of March, the southern province Noord-Brabant had about half of all detected infections in the Netherlands despite this province only accounting for 15 per cent of the Dutch population. At the same time, the northern provinces were almost free of infections. With regard to reported confirmed COVID-19 cases, hospitalizations and deaths, Noord-Brabant was leading in numbers per 100,000 residents as well as in terms of the proportion of total Dutch confirmed COVID-19 cases.

For a panel of individual employees, we apply a difference-in-difference specification at the weekly level by interacting the 0–1 indicator for 2020 (which is set to zero for 2019 observations) with 0–1 indicators for each of the first thirteen calendar weeks of the year. The interaction terms are used for a comparison of the outcome variable by calendar week relative to week 9–the week of the COVID-19 outbreak in the Netherlands. For each outcome variable, the specification is

1 $$ y_{it} = \alpha_{i} + \mathop \sum \limits_{\begin{subarray}{l} \tau = 1 \\ \tau \ne 9 \end{subarray} }^{13} \beta_{\tau } DW_{\tau } + \mathop \sum \limits_{\begin{subarray}{l} \tau = 1 \\ \tau \ne 9 \end{subarray} }^{13} \gamma_{\tau } DY_{c} \times DW_{\tau } + \delta DY_{c} + \eta \prime X_{ic} + \varepsilon_{it} $$

$$ \{ i \in 1, \ldots ,N;\,\,t \in 1, \ldots ,13\;\,\,\text {for} \, c = 2019;\;\,\,t \in 14, \ldots ,26\;\,\,\text {for} \, c = 2020\} $$

where *y* is the outcome variable; the subscripts *i*, *t* and *c* refer to individual, week and year, respectively. $\alpha$ is an individual fixed effect; $\tau$ represents the calendar week number; *DY* and *DW* are 0–1 indicator variables for year and calendar week. $\varepsilon$ is an idiosyncratic error term.

**Fig. 3:**
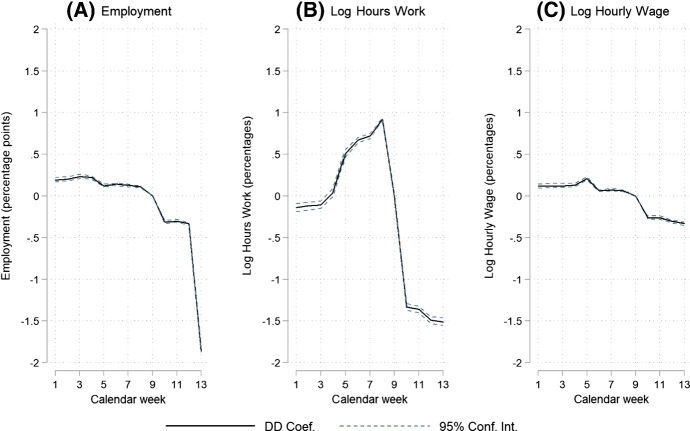
COVID-19 Difference-in-Difference (DD) effects on employment, log hours worked and log hourly wages (Eq. (1)) *Notes*: Parameter estimates of the double interaction terms between year and calendar week. Each graph represents a single regression for a different outcome variable. Reference year is 2019 and reference calendar week is 9. The 95% confidence intervals are computed based on standard errors clustered by individual. The total number of estimated parameters equals 75

The vector *X* contains 50 variables which are time constant within a year but may vary between the two years, and which is included to reduce the impact of any variation in observables by calendar year. *X* includes dummy variables for age (6 categories), job characteristics (type of contract (2), type of job (4), full-time/part-time status (2)).^5^ These variables are all measured in calendar week 9, preventing any endogeneity issues resulting from changes in covariates because of COVID-19. Additionally, *X* contains dummy variables for firm characteristics (size (3), economic sector (20) and a dummy variable for missing firm data although less than 1 per cent of observations fall in this category) and for household characteristics (married (1) and home location (11 provincial regions)), which are all measured on 31 December of the previous year. The results provided in Fig. 3 are robust to excluding $X$ and are available upon request.

Besides estimating baseline Eq. (1), we estimate a corresponding heterogeneous difference-in-difference equation to investigate the heterogeneity of the COVID-19 impacts. This model complements (1) by also including triple and double interactions between year, calendar week, and all variables in vector *X*^6^:

2 $$ \begin{aligned} y_{it} = \alpha_{i} + & \mathop \sum \limits_{\begin{subarray}{l} \tau = 1 \\ \tau \ne 9 \end{subarray} }^{13} \left[ {\beta_{\tau } DW_{\tau } + \gamma_{\tau } DY_{c} \times DW_{\tau } + (\kappa_{\tau } \prime X_{ic} )DW_{\tau } + (\lambda_{\tau } \prime X_{ic} ) \times DY_{c} \times DW_{\tau } } \right] \\ & + (\mu \prime X_{ic} ) \times DY_{c} + \delta DY_{c} + \eta \prime X_{ic} + \varepsilon_{it} \\ \end{aligned} $$

where $\kappa_{\tau }$and $\lambda_{\tau }$ and $\mu$ are additional parameters to be estimated, with vector $\lambda_{\tau }$ including the key parameters of interest.

## Data

We use administrative data from Statistics Netherlands. For computational reasons, we take a 50 per cent random sample of Dutch employees. We select two cohorts of employees who are followed from January until March. Specifically, we include employees who were employed in calendar week 9 of 2019 and we select employees who were employed in calendar week 9 of 2020. We follow individuals from January 1 until March 31, calendar week 13, of each calendar year. Table 1 reports the individual summary statistics for calendar week 9 of 2019 and 2020, respectively. Although there are some small differences in background characteristics, on the whole, the employee population in week 9 is quite similar in 2019 and 2020.

**Table 1:** Individual summary statistics by year (proportions unless otherwise noted)

	2019		2020	
	Mean	St. Dev	Mean	St. Dev
Employment	1	0	1	0
Work hours (log)	4.6926	0.6770	4.7009	0.6623
Work hours (monthly hours)	126.95	51.29	127.2959	50.6043
Hourly wage (log)	2.7902	0.5923	2.8226	0.5885
Hourly wage (euro per month)	19.06	13.65	19.6545	16.5013
Gross wage (log)	7.4762	1.0923	7.5190	1.0722
Gross wage (euro per month)	2618.73	2193.85	2703.85	2258.76
Female	0.4788	0.4995	0.4814	0.4997
Age				
14 $$ \le$$ age $$< $$ 20	0.0797	0.2708	0.0791	0.2700
20 $\le {\text{age }} <$ 25	0.0965	0.2952	0.0967	0.2955
25 $\le {\text{age }} <$ 35	0.2143	0.4103	0.2158	0.4113
35 $\le {\text{age }} <$ 45	0.1920	0.3939	0.1908	0.3929
45 $\le {\text{age }} <$ 55	0.2248	0.4175	0.2188	0.4134
55 $\le {\text{age }} <$ 60	0.1025	0.3033	0.1035	0.3047
60 $\le {\text{age}} <$ 70	0.0903	0.2866	0.0953	0.2937
Dutch	0.8729	0.3331	0.8671	0.3394
Partnered	0.6356	0.4813	0.6313	0.4825
Type of contract				
Permanent contract	0.6293	0.4830	0.6757	0.4681
Fixed contract	0.3425	0.4745	0.2962	0.4566
Other contract	0.0282	0.1655	0.0281	0.1654
Type of job				
Regular job	0.8293	0.3763	0.8054	0.3959
Flexible job	0.1104	0.3133	0.1341	0.3408
Payrolling job	0.0055	0.0739	0.0063	0.0792
Intern job	0.0169	0.1288	0.0169	0.1289
Full-time/part-time status				
$\ge$ 35 work hours a week	0.4837	0.4997	0.4829	0.4997
20 $\le$ hours a week $<$ 35	0.3146	0.4643	0.3218	0.4672
Hours a week $<$ 20	0.2017	0.4013	0.1952	0.3964
Province				
Groningen	0.0323	0.1768	0.0322	0.1766
Friesland	0.0352	0.1844	0.0352	0.1843
Drenthe	0.0269	0.1619	0.0269	0.1617
Overijssel	0.0680	0.2518	0.0682	0.2521
Flevoland	0.0250	0.1562	0.0253	0.1569
Gelderland	0.1202	0.3252	0.1203	0.3254
Utrecht	0.0808	0.2725	0.0810	0.2729
Noord-Holland	0.1661	0.3722	0.1661	0.3721
Zuid-Holland	0.2109	0.4080	0.2109	0.4080
Zeeland	0.0207	0.1425	0.0207	0.1423
Noord-Brabant	0.1520	0.3590	0.1520	0.3591
Limburg	0.0618	0.2408	0.0612	0.2397
Number of individuals (#)	3,848,057		3,893,467	

Sample means and standard deviations for individual characteristics are provided for calendar week 9 in 2019 and 2020, respectively. Summary statistics are not provided for all variables

## Results

**Fig. 4:**
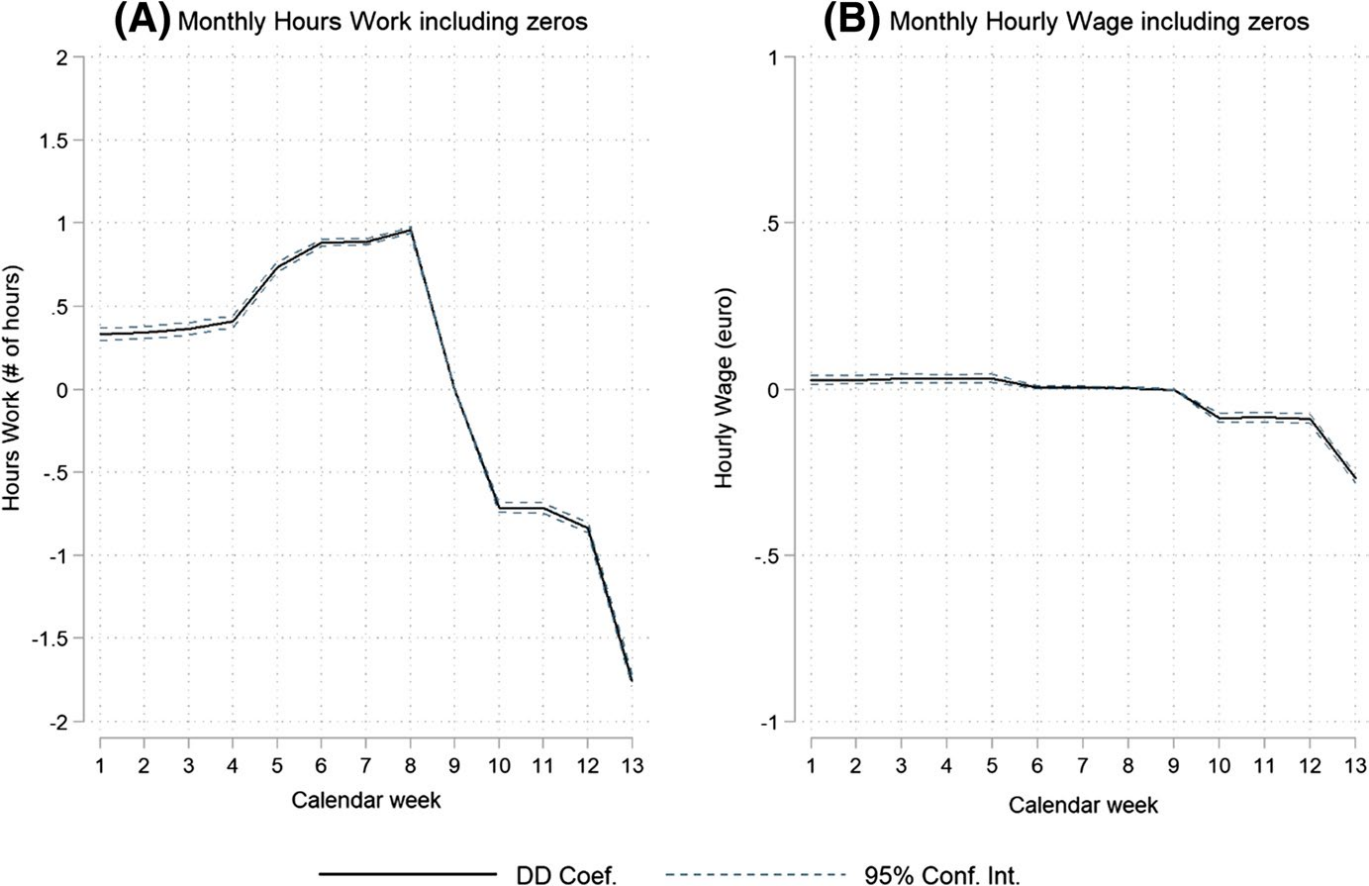
COVID-19 Difference-in-Difference (DD) effects on hours worked and hourly wages including zeros for the unemployed (Eq. (1)) *Notes*: Parameter estimates of the double interaction terms between year and calendar week. Each graph represents a single regression for a different outcome variable. Each outcome variable is in levels and zeros are used for unemployed individuals. Reference year is 2019 and reference calendar week is 9. The 95% confidence intervals are computed based on standard errors clustered by individual. The total number of estimated parameters equals 75

We report on three sets of novel results. First, the estimated week effects of Eq. (1) show that employment decreased slightly in weeks 10 to 12 and more substantially, by about 2 percentage points, in week 13 (Fig. 3). The evidence shows a slightly higher employment rate (0.1 to 0.2 percentage point) in weeks 1 to 8 in 2020 (relative to 2019). For the number of paid working hours, a comparable development is observed with a 1.5 per cent decrease in hours in week 13. We observe a small negative effect from COVID-19 on hourly wages of about 0.3 per cent.^7^ The reported COVID-19 effects on working hours and hourly wages are conditional on employment. The parallel trends restriction holds for the models on employment and hourly wages, but not for the model of working hours due to the fact that 2020 is a leap year. Including zeros for the unemployed, which limits the impact of selection into employment, Fig. 4 shows a reduction of 1.75 h in monthly working hours and a reduction of 0.25 euro in hourly wages. Relative to monthly mean working hours of 127 and a mean hourly wage of 19.65 in week 9 of 2020, this represents a decrease of 1.4 per cent and 1.3 per cent, respectively. The very small positive results for weeks 1 to 8 indicate that the labour market in 2020 was very similar to the labour market in 2019 before COVID-19 arrived; if anything, employment had been more stable for those employed in week 9 in 2020 than in 2019.

The effects of COVID-19 on working hours are consistent with, but somewhat smaller than, those reported by Von Gaudecker et al. (2020) as they find a reduction in total working hours of 11 per cent or 3 h. This difference in results could be explained by changes to respondents’ actual working hours whereas in our study employees’ paid working hours as per income statement could remain the same. Overall, the evidence suggests that employment and working hours are the relevant margins of labour adjustment rather than hourly wages in the first response to the COVID-19 shock.

**Fig. 5:**
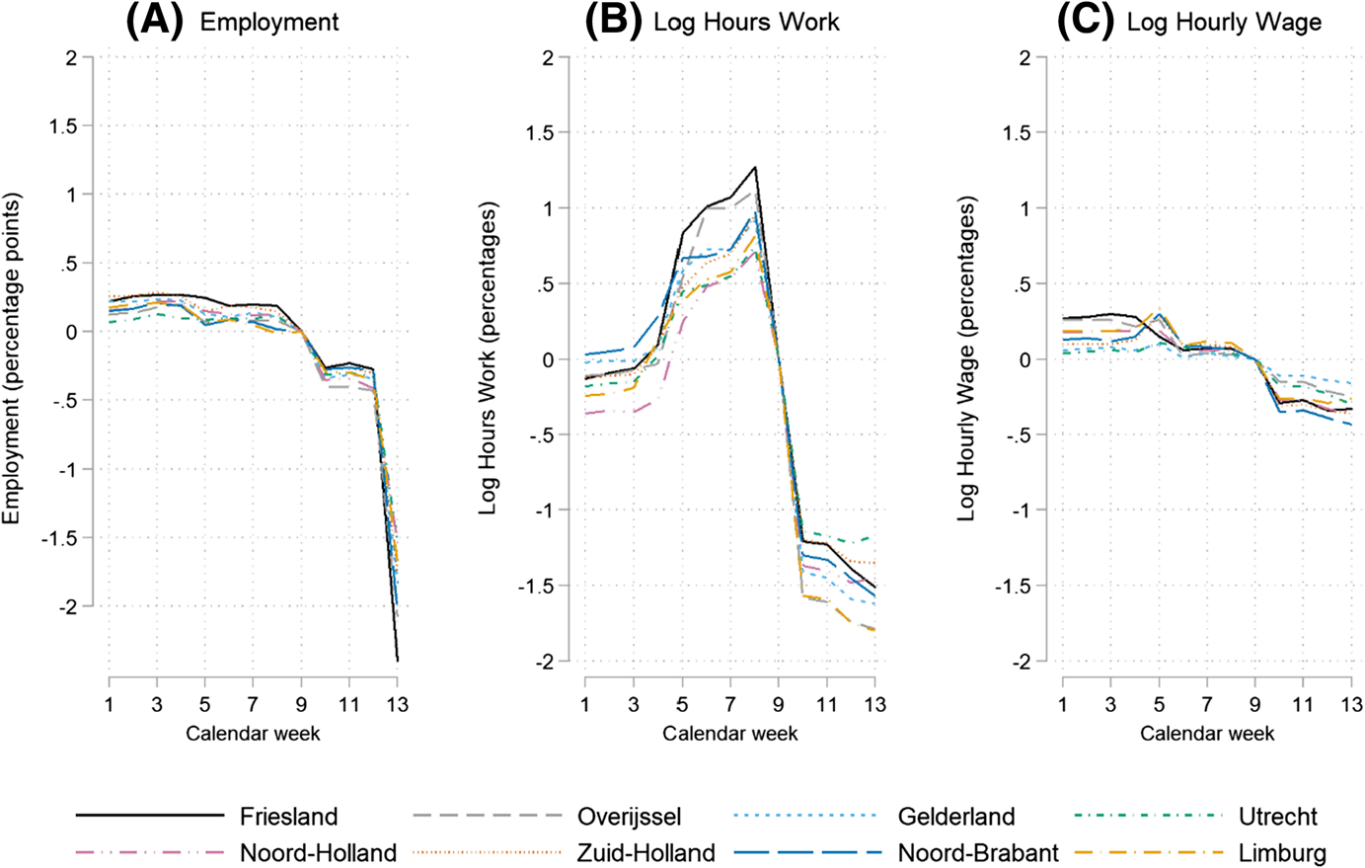
COVID-19 Difference-in-Difference effect stratified by province (Eq. (1)) *Notes*: Each graph represents a different outcome variable and each line represents a single regression for a different province. Several provinces are left out from Fig. 5 to ensure clear graphs. The figure for all provinces is available from the authors upon request

Second, separate estimation of Eq. (1) for each of the provinces indicate small regional differences in the changes in the outcome variables (Fig. 5). Importantly, these regional differences do not seem to be strongly related to the Dutch COVID-19 hotspot provincial areas of March 2020.

Third, Table 2 presents the estimated parameters on the triple difference interactions of Eq. (2) revealing which categories of employees had the strongest decline in the three outcome variables in week 13 of 2020. Consistent with the results provided in Fig. 5, the evidence in Table 2 does not suggest a region-specific impact of COVID-19 on the outcome variables when comparing COVID-19 hotspots such as Noord-Brabant and Limburg to other Dutch provinces, including some of the northern provinces such as Groningen and Friesland which had very few cases but experienced a larger negative impact on employment than Noord-Brabant. Other characteristics of employees are shown to be more relevant. Individuals who (i) are aged below 20 years, (ii) have a non-permanent contract; and (iii) are in a flexible or payrolling job, were most negatively affected by the economic effects of the COVID-19 shock. Overall, for a country with a relatively small area size like the Netherlands, the results suggest that the employee’s job characteristics are more important than the regional location of residency for the effects of COVID-19 on individual labour market outcomes.

**Table 2:** The role of observed individual characteristics in the effects of COVID-19 (Eq. (2))

	Employment (= 1) (1)	Working hours (log) (2)	Hourly wage (log) (3)
*Triple interaction term:* $DY\times D{W}_{13}\times$			
$\mathrm{FEMALE}:$ relative to $\mathrm{ male}$	0.0008** (0.0003)	− 0.0019*** (0.0006)	0.0006** (0.0003)
AGE: $\mathrm{ relative to }14\le \mathrm{AGE}<20$ yrs			
$20\le \mathrm{AGE}<25\mathrm{ years}$	0.0303*** (0.0010)	0.0269*** (0.0022)	− 0.0017** (0.0008)
$25\le \mathrm{AGE}<35\mathrm{ years}$	0.0250*** (0.0010)	0.0455*** (0.0020)	0.0014* (0.0008)
$35\le \mathrm{AGE}<45\mathrm{ years}$	0.0223*** (0.0010)	0.0475*** (0.0020)	0.0027*** (0.0008)
$45\le \mathrm{AGE}<55$ years	0.0217*** (0.0010)	0.0476*** (0.0020)	0.0027*** (0.0008)
$55\le \mathrm{AGE}<60\mathrm{ years}$	0.0210*** (0.0010)	0.0474*** (0.0020)	0.0030*** (0.0008)
$60\le \mathrm{AGE}<70\mathrm{ years}$	0.0171*** (0.0010)	0.0497*** (0.0021)	0.0029*** (0.0008)
$\mathrm{NON}$-$\mathrm{DUTCH NATIONALITY}$ : relative to $\mathrm{ Dutch}$	− 0.0032*** (0.0004)	− 0.0073*** (0.0008)	− 0.0011*** (0.0003)
PARTNERED: relative to $\mathrm{ no partner}$	− 0.0009*** (0.0003)	0.0016*** (0.0005)	0.0003 (0.0002)
CONTRACT: relative to $\mathrm{ permanent contract}$			
$\mathrm{FIXED CONTRACT}$ :	−0.0254*** (0.0004)	0.0024*** (0.0007)	0.0004 (0.0003)
$\mathrm{OTHER CONTRACT}$	−0.0182*** (0.0005)	0.0128*** (0.0007)	−0.0060 (0.0005)
TYPE OF JOB: relative to $\mathrm{ regular job}$			
$\mathrm{FLEXIBLE JOB}$	−0.0628*** (0.0007)	−0.0445*** (0.0015)	0.0012** (0.0005)
$\mathrm{PAYROLLING JOB}$	−0.1266*** (0.0036)	−0.0007 (0.0075)	0.0116*** (0.0025)
$\mathrm{INTERN JOB}$	0.0095*** (0.0013)	−0.0176*** (0.0030)	0.0075*** (0.0021)
$\mathrm{PROVINCE}$ : relative to $\mathrm{ Noord}$-$\mathrm{Brabant}$			
$\mathrm{GRONINGEN}$	−0.0018** (0.0008)	0.0017 (0.0015)	0.0003 (0.0006)
$\mathrm{FRIESLAND}$	−0.0029*** (0.0007)	0.0014 (0.0014)	0.0005 (0.0006)
$\mathrm{DRENTHE}$	−0.0046*** (0.0008)	0.0009 (0.0016)	−0.0006 (0.0007)
$\mathrm{OVERIJSSEL}$	−0.0003 (0.0006)	−0.0010 (0.0011)	0.0015*** (0.0005)
$\mathrm{FLEVOLAND}$	0.0034*** (0.0009)	0.0033*** (0.0016)	−0.0006 (0.0007)
$\mathrm{GELDERLAND}$	0.0013*** (0.0005)	0.0004 (0.0009)	0.0023*** (0.0004)
$\mathrm{UTRECHT}$	0.0029*** (0.0005)	0.0041*** (0.0010)	0.0009** (0.0004)
$\mathrm{NOORD}$-$\mathrm{HOLLAND}$	0.0040*** (0.0004)	0.0036*** (0.0008)	0.0006* (0.0004)
$\mathrm{ZUID}$-$\mathrm{HOLLAND}$	0.0020*** (0.0004)	0.0025*** (0.0008)	0.0002 (0.0003)
$\mathrm{ZEELAND}$	−0.0041*** (0.0009)	−0.0023 (0.0018)	0.0011 (0.0007)
$\mathrm{LIMBURG}$	0.0032*** (0.0006)	−0.0018 (0.0011)	0.0012*** (0.0005)
Number of individuals	4,211,030	4,211,010	4,204,164
Number of observations	100,639,812	98,674,164	98,309,619

## Conclusion

Our study examined the impact of the COVID-19 pandemic in the Netherlands in the initial month after the outbreak, analysing the role of coronavirus hotspot areas in the impact on local labour markets. There are two major outcomes. First, we find that employment decreased by 2 percentage points in March 2020, which is a relatively low reduction compared to studies from other countries. However, the stronger negative effects in other research were estimated using information covering a longer period of time after the COVID-19 outbreak commenced.

Second, the results in this paper indicate limited regional differences in the negative impacts on the Dutch labour market during the outbreak of COVID-19. It appears that higher virus case numbers did not reinforce the decline of the labour market beyond the impacts from the government-enforced lockdown. As a result, the northern Dutch provinces, which experienced a limited number of COVID-19 cases, suffered a similar (or even worse) decline in labour market conditions, as compared with the provinces that were severely affected by the virus.

We examined the net outcome of employment, but did not disentangle the supply and demand side impacts in the labour market. Neither did we exploit any sectoral specialization in economic activities across regions. These factors could potentially contribute to a (partial) explanation of the within-country differences in COVID-19 impacts on regional labour markets, but investigation of these is outside the scope of this paper.

The outcome of an absence of regional differences in the first month of the outbreak suggests that policy makers should be cautious when implementing preventive measures nationwide as the economic costs can be substantial. Thus, where feasible, preventive health measures should be at the regional level, isolating hotspots from low-risk areas. This would allow relatively unaffected parts of the country to continue economic activities as much as possible, ultimately benefitting the nation as a whole.

## Footnotes

1 See Hoekman, Smits, and Koolman (2020) for an overview of health policy measures during the first months of the COVID-19 pandemic in the Netherlands. See Campbell Collaboration (2020) and Mayhew and Anand (2020) for a systematic review of relevant policy response to the COVID-19 crisis. See Baldwin and di Mauro (2020) and Brodeur, Gray, Islam and Bhuiyan (2020) for comprehensive reviews of the economic consequences of the COVID-19 pandemic.

2 Other studies, that focussed on different labour-market aspects, demonstrated substantial changes in the labour market using information on worker and unemployment flows in the initial months (Germany – Bauer and Weber, 2020; the Nordic countries – Juranek, Paetzold, Winner and Zoutman, 2020; Italy – Casarico and Lattanzio, 2020). There are also studies that document decreases in the posting of vacancies (US – Forsythe, Kahn, Lange, and Wiczer, 2020; Austria – Bamieh and Ziegler, 2020; Sweden – Hensvik, Le Barbanchon, and Rathelot, 2021).

3 There are additional spatial papers focusing on the health aspects of COVID-19. Menon (2021) relates the spatial variation of COVID-19 in India to individual characteristics such as the body mass index. Di Porto, Naticchioni, and Scrutinio, (2020) exploited that Italy (like many other countries) had a less stringent lockdown for essential economic sectors. They found that an additional 100 essential workers per square kilometre resulted in an additional 0.25 daily COVID-19 cases.

4 Logarithms are used to focus on percentage changes. The estimated effects are robust to a specification in levels.

5 The number of categories does not include the reference category.

6 $X$ is the same as in Eq. (1), but here we also include triple and double interaction terms between year, calendar week and the individual’s gender and nationality, which are time constant and absorbed by the individual fixed effects in (1).

7 As the data on working hours and wages are reported at the monthly level, a small increase in 2020 relative to 2019 can be observed for working hours, explained by the fact that 2020 is a leap year. For the hourly wage, the impact of the leap year is removed as monthly gross wage is divided by monthly number of working hours.
